# MicroRNAs in the development and neoplasia of the mammary gland

**DOI:** 10.12688/f1000research.12005.2

**Published:** 2017-10-03

**Authors:** Manoj Kumar Jena

**Affiliations:** 1 Department of Biotechnology, School of Bioengineering and Biosciences, Lovely Professional University (LPU), Phagwara, Punjab, 144411, India

**Keywords:** Mammary gland, miRNA, development, lactation, neoplasia

## Abstract

Study on the role of microRNAs (miRs) as regulators of gene expression through posttranscriptional gene silencing is currently gaining much interest,due to their wide involvement in different physiological processes. Understanding mammary gland development, lactation, and neoplasia in relation to miRs is essential. miR expression profiling of the mammary gland from different species in various developmental stages shows their role as critical regulators of development. miRs such as miR-126, miR-150, and miR-145 have been shown to be involved in lipid metabolism during lactation. In addition, lactogenic hormones influence miR expression as evidenced by overexpression of miR-148a in cow mammary epithelial cells, leading to enhanced lactation. Similarly, the miR-29 family modulates lactation-related gene expression by regulating DNA methylation of their promoters. Besides their role in development, lactation and involution, miRs are responsible for breast cancer development. Perturbed estrogen (E2) signaling is one of the major causes of breast cancer. Increased E2 levels cause altered expression of ERα, and ERα-miR cross-talk promotes tumour progression. miRs, such as miR-206, miR-34a, miR-17-5p, and miR-125 a/b are found to be tumour suppressors; whereas miR-21, miR-10B, and miR-155 are oncogenes. Oncogenic miRs like miR-21, miR-221, and miR-210 are overexpressed in triple negative breast cancer cases which can be diagnostic biomarker for this subtype of cancer.  This review focuses on the recent findings concerning the role of miRs in developmental stages of the mammary gland (mainly lactation and involution stages) and their involvement in breast cancer progression. Further studies in this area will help us to understand the molecular details of mammary gland biology, as well as miRs that could be therapeutic targets of breast cancer.

## Introduction

MicroRNAs (miRs) are small, endogenous noncoding RNAs that regulate gene expression post-transcription, by degrading target mRNA or repressing translation. They are hardy in nature, being resistant to RNAse degradation, acidic pH, and repeated freezing and thawing, and are stable at room temperature ^1^. Matured miRs integrate into the RNA - induced silencing complex (RISC) and direct it to target mRNAs through partial sequence complementarity. miRs down regulate the target gene expression by translation repression (48% through RISC complex), or mRNA degradation (29%), or by both phenomena (23%) ^2^. In addition to their involvement in many other physiological processes, they play crucial roles in mammary gland development and lactation, and their deregulation can lead to breast cancer ^3^. This review provides an overview on the recent findings concerning the role of miRs in the developmental stages of the mammary gland and breast cancer progression.

## miRs in mammary glands

The study of miRs in mammary gland development is essential, as miRs in milk are derived from the mammary gland and can be a biomarker of a healthy lactating gland, as well as protecting infants and promoting their development ^4^.

miRs in milk during lactation are putative markers of activities in mammary gland and act as functional signals for proper growth and development of the young. The highly abundant miRs in milk during lactation cycle are miR-191, 184, 181, 148, 375, and miRs of let-7 family (7f, 7a and 7i) as evidenced from studies in the marsupial tammar wallaby ^5^. Comparative analysis of the miRNome of bovine milk, mammary tissue, milk fat, and whey revealed 188 miRs in common, with some novel miRs discovered ^6^. The miR signature of milk fat was similar to that of mammary gland tissue miRs. Functional studies of highly expressed miRs in those fractions indicated their role as regulators of mammary gland functions contributing to healthy milk production ^6^.

## miRs controlling lactation

It was observed that miR-126 plays a role in mammary epithelial cells (MECs) by modulating lipid synthesis ^7^. *FASN* (fatty acid synthase) gene expression is increased when miR-126-3p is inhibited, suggesting its involvement in lipid metabolism in the mammary gland. In addition, estradiol and progesterone enhanced lipid synthesis by downregulating the levels of miR-126-3p ^7^. Similarly, miR-150 hampers lipogenesis in MECs and reduces secretory activity ^8^, while miR-145 facilitates milk fat synthesis in lactating goats by targeting the gene *INSIG1* (insulin induced gene 1) ^9^.

The role that miRs play as regulators of lipogenesis was confirmed by overexpression studies, which showed that there was more synthesis of fat droplets, accumulating triacylglycerols, and a higher proportion of unsaturated fatty acids in lactating MECs. For instance, miR-24 was found to be expressed at a much higher level during peak lactation in goats and affects triacylglycerol content, unsaturated fatty acid concentration, and expression of target genes such as *FASN*, *SREBF1* (sterol regulatory element binding transcription factor1), *SCD* (stearoyl-CoA desaturase), *GPAM* (glycerol-3-phosphate acyltransferase; mitochondrial), and *ACACA* (acetyl-CoA carboxylase) ^10^.

miR expression in MECs is regulated by lactogenic hormones (dexamethasone, insulin, and prolactin) as evidenced by an increased miR-148a level in bovine MECs, which is probably associated with increased milk production during lactation in cows ^11^. The miR-29 family affects lactation by regulating the DNA methylation of target genes *DNMT3A* and *DNMT3B* (DNA (cytosine-5)-methyltransferase 3A and 3B) in MECs of dairy cows ^12^. Moreover, inhibition of the miR-29 family resulted in hypermethylation of promoters of lactation–related genes leading to decreased secretion of triglycerides, proteins, and lactose by the epithelial cells. miR-486 facilitates lactation by downregulating *PTEN* (phosphatase and tensin homolog) target gene. Downregulation of this gene affects the expression of downstream genes, such as *AKT*, *mTOR* and *β-casein*, which have crucial roles in mammary gland development and lactation ^13^. Additionally, diet restriction has been shown to affect the miRNome of lactating mammary glands, indicating the role of miRs in regulating milk composition ^14^. Lin *et al.* (2013) ^15^ observed group of miRs (such as miR-23a, 27b, 103, and 200a) affect milk fat synthesis with synergistic action. miR profiling of lactating and nonlactating bovine mammary gland revealed miR-125b, 181a, and 199b expression level reduced in non-lactation period; whereas miR-141, 484, and 500 expression level was higher in lactation period ^16^. Highly expressed miRs such as let-7f-5p is found to target many genes involved in protein, carbohydrate, and triglyceride synthesis. The human milk enriched miR such as miR-22-3P regulates development and differentiation of T-lymphocytes. The miR-181a-5p and miR-182-5p have crucial role in immune cell differentiation, miR-375 is required for glucose homeostasis, and miR-148a-3p is a tumour suppressor and involved in liver development ^17^.

## miRs controlling involution and breast cancer

Identification and characterization of miRs involved in breast cancer will facilitate targeting miRs for possible therapy. Improper involution possibly contributes towards tumour development ^18^. miR-424(322)/503 regulates mammary involution in humans by targeting *BCL-2* (B-cell lymphoma 2; anti-apoptotic) and *IGF1R* (insulin like growth factor-1-receptor) genes. The loss of this miR leads to malignancy and nonresponse to chemotherapy, demonstrating its role as a tumour suppressor ^18^. By contrast, some miRs, such as miR-660-5p, promote tumour development and metastasis, and the level of miR-660-5p was found to be increased in MCF7 breast cancer cell lines ^19^. This miR’s tumour promoting activity was confirmed by observation of reduced invasion of MCF7 cells at reduced miR-660-5p levels.

One of the major causes of breast cancer is disturbed estrogen signaling, where the altered expression of estrogen receptor α (ERα) and its cross-talk with the related miR culminates in neoplasia ^20^. Studies on the effect of E2 on the expression pattern of miRs in MCF7 and ZR75 cell lines revealed 172 miRs that were up or downregulated. Notable miRs are miR-206, miR-34a, miR-17-5p, and miR-125 a/b, which act as tumour suppressors, and miR-21, miR-10B, and miR-155, which act as oncogenes ^21^. Another study using an ACI rat model for the effect of E2 on the miR signature showed 33 dysregulated miRs ^22^. Additionally, the use of ellagic acid (natural phenol antioxidant) reversed the dysregulation of miR-206, miR-182, miR-375, miR-127, miR-183, and miR-122, subsequently modulating the target proteins ERα, RASD1, cyclin D1, FoxO1, FoxO3a, Bcl-w, Bcl-2 and cyclin G118 ^22^. Furthermore, overexpression of the tumour suppressing miR-133a in MCF-7 and MDA-MB-231 cells suppressed phosphorylated Akt (p-Akt) protein and inhibited p-Akt nuclear translocation, and this miR also regulates the cell cycle of cancerous cells by targeting the *EGFR* (epidermal growth factor receptor) gene ^23^.

miR-206 is found to play a crucial role in *BRCA1* (Breast CAncer susceptibility gene; tumour suppressor) depleted mouse mammary gland ^24^. Overexpression of miR-206 showed no effect on lactation, but did have a role in tissue remodeling through increasing fat tissue and reducing branching morphogenesis. There may be a possibility of increased miR-206 levels due to *BRCA1* loss, culminating in mammary gland remodeling and tumour development ^24^. miR-184 is found to be a tumour suppressor by regulating the number of genes in the PI3K/AKT/mTOR pathway, as observed by miR profiling of the pubertal mouse mammary gland ^25^. This pathway is important in mammary gland development and lactation ^26^. miR-184 is only expressed in epithelial cells, and the level increases during differentiation of cells from terminal end bud into ductal epithelial cells ^25^. The miR 126/126* pair is observed to repress the recruitment of mesenchymal stem cells and inflammatory monocytes into the tumour stroma, thus inhibiting breast cancer metastasis ^27^. Urinary miR detection of overexpressed miR-155, and lowered expression of miR – 21, 125b, and 451 can act as non-invasive biomarker for breast cancer detection ^28^. Chan *et al.* (2013) ^29^ identified the miR signature (miR-1, 92a, 133a, and 133b) in serum which can be used as biomarkers for breast cancer detection as a noninvasive diagnostic strategy. Similar study by Mar-Aguilar *et al.*, (2013) ^30^ identified miR-145, 155, and 382 as potential biomarker for breast cancer detection. Deregulated expression of miR-17, 34a, 155 ,and 373 and their concentration in serum as cell-free miR probably connects to the progression and metastasis of breast cancer ^31^.

Exosomes are cell - derived small vesicles (40 – 100 nm) containing mRNA, protein, miR etc. They are crucial mediator of inter cellular signaling during cancer development. Exosomes derived from cancer associated fibroblasts in breast cancer have 3 miRs such as miR-21, -143, and -378e which promote stemness, epithelial-mesenchymal transition, and anchorage-independent cell growth, thus accelerating oncogenic signaling in breast cancer cells ^32^. The exosomes released from cancer cells of breast cancer patients induce the non-tumorigenic epithelial cells to form tumors with the help of dicer endonuclease ^33^.

Triple negative breast cancer (TNBC) is a subtype of breast cancer where the cancer cells lack the estrogen receptor, progesterone receptor, and human epidermal growth factor receptor 2 (HER2) ^34^. Out of total breast cancer cases 10 – 20% are TNBC cases and various studies showed the expression of specific miR pattern in these cases which can act as biomarker for cancer diagnosis ^30, 35, 36^. A recent study on paired sera and tissue samples of TNBC patients in India revealed overexpression of 3 oncogenic miRs (miR – 21, 221, and 210) and down regulation of miR – 195 and miR – 145 as compared to that of triple positive breast cancer cases. The TNBC cases were found to be highly prevalent (73.9%) in pre-maunopausal (<35 yr) women and the miR let7a was overexpressed although it is known as a tumour suppressor. Increased serum level of exosomal miR-373 can also be a marker for TNBC cases ^37^. The study of TNBCs with lymphnode metastases showed association of 27 miRs with metastatic capability. This study observed 71 differentially expressed miRs in TNBCs including miR-200 family and miR-17-92 oncogenic cluster ^38^.

## Conclusions

miRs have been shown to be one of the major regulators of mammary gland development and neoplasia. miRNome studies of mammary gland in different developmental stages and cancerous tissues will elucidate biomarkers for early cancer diagnosis, and may be used as therapeutic targets. Future studies focusing on the cross-talk between miRs and target genes with the signaling pathways involved in development and neoplasia of mammary gland will open the door to understand mammary gland biology and oncogenesis in molecular detail.

## References

[1] Gigli I Maizon DO: microRNAs and the mammary gland: A new understanding of gene expression. *Genet Mol Biol.* 2013;36(4):465–474. 10.1590/S1415-47572013005000040 24385846 PMC3873174

[2] Jin HY Xiao C: MicroRNA mechanisms of action: What have we learned from mice? *Front Genet.* 2015;6:328. 10.3389/fgene.2015.00328 26635864 PMC4644800

[3] Wang CD Long K Jin L: Identification of conserved microRNAs in peripheral blood from giant panda: expression of mammary gland-related microRNAs during late pregnancy and early lactation. *Genet Mol Res.* 2015;14(4):14216–14228. 10.4238/2015.November.13.5 26600479

[4] Alsaweed M Lai CT Hartmann PE: Human milk miRNAs primarily originate from the mammary gland resulting in unique miRNA profiles of fractionated milk. *Sci Rep.* 2016;6:20680. 10.1038/srep20680 26854194 PMC4745068

[5] Modepalli V Kumar A Hinds LA: Differential temporal expression of milk miRNA during the lactation cycle of the marsupial tammar wallaby ( *Macropus eugenii*). *BMC Genomics.* 2014;15:1012. 10.1186/1471-2164-15-1012 25417092 PMC4247635

[6] Li R Dudemaine PL Zhao X: Comparative Analysis of the miRNome of Bovine Milk Fat, Whey and Cells. *PLoS One.* 2016;11(4):e0154129. 10.1371/journal.pone.0154129 27100870 PMC4839614

[7] Chu M Zhao Y Feng Y: MicroRNA-126 participates in lipid metabolism in mammary epithelial cells. *Mol Cell Endocrinol.* 2017;454:77–86. pii: S0303-7207(17)30309-X, In press. 10.1016/j.mce.2017.05.039 28599789

[8] Heinz RE Rudolph MC Ramanathan P: Constitutive expression of microRNA-150 in mammary epithelium suppresses secretory activation and impairs *de novo* lipogenesis. *Development.* 2016;143(22):4236–4248. 10.1242/dev.139642 27729410 PMC5117217

[9] Wang H Shi H Luo J: MiR-145 Regulates Lipogenesis in Goat Mammary Cells Via Targeting *INSIG1* and Epigenetic Regulation of Lipid-Related Genes. *J Cell Physiol.* 2017;232(5):1030–1040. 10.1002/jcp.25499 27448180

[10] Wang H Luo J Chen Z: MicroRNA-24 can control triacylglycerol synthesis in goat mammary epithelial cells by targeting the fatty acid synthase gene. *J Dairy Sci.* 2015;98(12):9001–14. 10.3168/jds.2015-9418 26476938

[11] Muroya S Hagi T Kimura A: Lactogenic hormones alter cellular and extracellular microRNA expression in bovine mammary epithelial cell culture. *J Anim Sci Biotechnol.* 2016;7:8. 10.1186/s40104-016-0068-x 26889380 PMC4756532

[12] Bian Y Lei Y Wang C: Epigenetic Regulation of miR-29s Affects the Lactation Activity of Dairy Cow Mammary Epithelial Cells. *J Cell Physiol.* 2015;230(9):2152–63. 10.1002/jcp.24944 25656908

[13] Li D Xie X Wang J: MiR-486 regulates lactation and targets the PTEN gene in cow mammary glands. *PLoS One.* 2015;10(3):e0118284. 10.1371/journal.pone.0118284 25738494 PMC4349860

[14] Mobuchon L Marthey S Le Guillou S: Food Deprivation Affects the miRNome in the Lactating Goat Mammary Gland. *PLoS One.* 2015;10(10):e0140111. 10.1371/journal.pone.0140111 26473604 PMC4608672

[15] Lin X Luo J Zhang L: MicroRNAs synergistically regulate milk fat synthesis in mammary gland epithelial cells of dairy goats. *Gene Expr.* 2013;16(1):1–13. 10.3727/105221613X13776146743262 24397207 PMC8750411

[16] Li Z Liu H Jin X: Expression profiles of microRNAs from lactating and non-lactating bovine mammary glands and identification of miRNA related to lactation. *BMC Genomics.* 2012;13:731. 10.1186/1471-2164-13-731 23270386 PMC3551688

[17] Alsaweed M Lai CT Hartmann PE: Human Milk Cells and Lipids Conserve Numerous Known and Novel miRNAs, Some of Which Are Differentially Expressed during Lactation. *PLoS One.* 2016;11(4):e0152610. 10.1371/journal.pone.0152610 27074017 PMC4830559

[18] Rodriguez-Barrueco R Nekritz EA Bertucci F: miR-424(322)/503 is a breast cancer tumor suppressor whose loss promotes resistance to chemotherapy. *Genes Dev.* 2017;31(6):553–566. 10.1101/gad.292318.116 28404630 PMC5393051

[19] Shen Y Ye YF Ruan LW: Inhibition of miR-660-5p expression suppresses tumor development and metastasis in human breast cancer. *Genet Mol Res.* 2017;16(1):gmr16019479. 10.4238/gmr16019479 28252173

[20] Manavathi B Dey O Gajulapalli VN: Derailed estrogen signaling and breast cancer: an authentic couple. *Endocr Rev.* 2013;34(1):1–32. 10.1210/er.2011-1057 22947396 PMC3565105

[21] Ferraro L Ravo M Nassa G: Effects of oestrogen on microRNA expression in hormone-responsive breast cancer cells. *Horm Cancer.* 2012;3(3):65–78. 10.1007/s12672-012-0102-1 22274890 PMC10358138

[22] Munagala R Aqil F Vadhanam MV: MicroRNA 'signature' during estrogen-mediated mammary carcinogenesis and its reversal by ellagic acid intervention. *Cancer Lett.* 2013;339(2):175–84. 10.1016/j.canlet.2013.06.012 23791885 PMC3775863

[23] Cui W Zhang S Shan C: microRNA-133a regulates the cell cycle and proliferation of breast cancer cells by targeting epidermal growth factor receptor through the EGFR/Akt signaling pathway. *FEBS J.* 2013;280(16):3962–74. 10.1111/febs.12398 23786162

[24] Wronski A Sandhu GK Milevskiy MJ: MicroRNA-206 is differentially expressed in *Brca1*-deficient mice and regulates epithelial and stromal cell compartments of the mouse mammary gland. *Oncogenesis.* 2016;5:e218. 10.1038/oncsis.2016.27 27043663 PMC4848838

[25] Phua YW Nguyen A Roden DL: MicroRNA profiling of the pubertal mouse mammary gland identifies miR-184 as a candidate breast tumour suppressor gene. *Breast Cancer Res.* 2015;17:83. 10.1186/s13058-015-0593-0 26070602 PMC4504458

[26] Wang Z Hou X Qu B: *Pten* regulates development and lactation in the mammary glands of dairy cows. *PLoS One.* 2014;9(7):e102118. 10.1371/journal.pone.0102118 25009983 PMC4092105

[27] Zhang Y Yang P Sun T: miR-126 and miR-126* repress recruitment of mesenchymal stem cells and inflammatory monocytes to inhibit breast cancer metastasis. *Nat Cell Biol.* 2013;15(3):284–94. 10.1038/ncb2690 23396050 PMC3672398

[28] Erbes T Hirschfeld M Rücker G: Feasibility of urinary microRNA detection in breast cancer patients and its potential as an innovative non-invasive biomarker. *BMC Cancer.* 2015;15:193. 10.1186/s12885-015-1190-4 25886191 PMC4383066

[29] Chan M Liaw CS Ji SM: Identification of circulating microRNA signatures for breast cancer detection. *Clin Cancer Res.* 2013;19(16):4477–87. 10.1158/1078-0432.CCR-12-3401 23797906

[30] Mar-Aguilar F Mendoza-Ramírez JA Malagón-Santiago I: Serum circulating microRNA profiling for identification of potential breast cancer biomarkers. *Dis markers.* 2013;34(3):163–9. 10.3233/DMA-120957 23334650 PMC3810231

[31] Eichelser C Flesch-Janys D Chang-Claude J: Deregulated serum concentrations of circulating cell-free microRNAs miR-17, miR-34a, miR-155, and miR-373 in human breast cancer development and progression. *Clin Chem.* 2013;59(10):1489–96. 10.1373/clinchem.2013.205161 23748853

[32] Donnarumma E Fiore D Nappa M: Cancer-associated fibroblasts release exosomal microRNAs that dictate an aggressive phenotype in breast cancer. *Oncotarget.* 2017;8(12):19592–19608. 10.18632/oncotarget.14752 28121625 PMC5386708

[33] Melo SA Sugimoto H O'Connell JT: Cancer exosomes perform cell-independent microRNA biogenesis and promote tumorigenesis. *Cancer Cell.* 2014;26(5):707–21. 10.1016/j.ccell.2014.09.005 25446899 PMC4254633

[34] Yang Q Liu HY Liu D: Ultrasonographic features of triple-negative breast cancer: a comparison with other breast cancer subtypes. *Asian Pac J Cancer Prev.* 2014;16(8):3229–32. 10.7314/APJCP.2015.16.8.3229 25921124

[35] Medimegh I Omrane I Privat M: MicroRNAs expression in triple negative vs non triple negative breast cancer in Tunisia: interaction with clinical outcome. *PLoS One.* 2014;9(11):e111877. 10.1371/journal.pone.0111877 25369070 PMC4219794

[36] Thakur S Grover RK Gupta S: Identification of Specific miRNA Signature in Paired Sera and Tissue Samples of Indian Women with Triple Negative Breast Cancer. *PLoS One.* 2016;11(7):e0158946. 10.1371/journal.pone.0158946 27404381 PMC4942139

[37] Eichelser C Stückrath I Müller V: Increased serum levels of circulating exosomal microRNA-373 in receptor-negative breast cancer patients. *Oncotarget.* 2014;5(20):9650–63. 10.18632/oncotarget.2520 25333260 PMC4259427

[38] Avery-Kiejda KA Braye SG Mathe A: Decreased expression of key tumour suppressor microRNAs is associated with lymph node metastases in triple negative breast cancer. *BMC cancer.* 2014;14(1):51. 10.1186/1471-2407-14-51 24479446 PMC3912930

