# Supplementary material for: MicroRNAs in the development and neoplasia of the mammary gland
Source: F1000Res. 2017 Oct 3;6:1018. Originally published 2017 Jun 28. [Version 2] doi: 10.12688/f1000research.12005.2 (PMC5609084; doi:10.12688/f1000research.12005.2)
Supplement: Peer review history

# Peer review: Referee response for version 2

**DOI:** 10.5256/f1000research.13851.r26603

**Published:** 2017-10-31

**Authors:** Mohammed Alsaweed

**Affiliations:** 1 College of Applied Medical Sciences, Majmaah University, Al Majma'ah, Saudi Arabia

All the suggestions made by the referee are sufficient and the paper is suggested to be indexed in F1000Research.

I have read this submission. I believe that I have an appropriate level of expertise to confirm that it is of an acceptable scientific standard.

# Peer review: Referee response for version 1

**DOI:** 10.5256/f1000research.12984.r24742

**Published:** 2017-09-20

**Authors:** Bhudev C. Das

**Affiliations:** 1 Molecular Oncology Laboratory, B.R. Ambedkar Centre for Biomedical Research (ACBR), University of Delhi, New Delhi, India

Review article on “MicroRNAs in the development and neoplasia of the mammary gland” submitted by M.K. Jena deals with how various microRNAs that regulate gene expression through post transcriptional gene silencing are important during development and progression as well as prognosis of cancer of the mammary gland in women. Though I understand it is a mini-review but it has not been written in a very focused and systematic way. There are huge numbers of publications in this field, particularly the role of mRNA in breast carcinogenesis. However, the author cites only 22 references in this article which is highly inadequate to justify the review on the role of miRNA in mammary gland development and carcinogenesis. Author must consult recently published literatures including a recent paper by Thakur et al. (2016) ^1^ and many of the references therein. There are a lot of sweeping comments have been made which indicates that the author has nothing much to do with cancer specialty.  The comments in abstract say ‘miRs are responsible for breast cancer development’. It is not straight forward and clear that miRNA are responsible of breast cancer development. They may be associated with but not directly correlated with cancer development. Similar sweeping comments are many in the text and these need to be addressed properly and the manuscript needs to be elaborative and focused. The presentation of the review article should be made with proper sub-headings on mammary gland development, lactation and carcinogenesis. 

 In summary, I suggest that the article can be considered for indexing only after satisfactory revision.

I have read this submission. I believe that I have an appropriate level of expertise to confirm that it is of an acceptable scientific standard, however I have significant reservations, as outlined above.

## References

[1] ThakurSGroverRKGuptaSYadavAKDasBC: Identification of Specific miRNA Signature in Paired Sera and Tissue Samples of Indian Women with Triple Negative Breast Cancer. *PLoS One*.2016;11(7) : 10.1371/journal.pone.0158946 e0158946 10.1371/journal.pone.0158946 27404381PMC4942139

# Response

**Published:** 2017-10-01

**Authors:** Manoj Jena

**Affiliations:** Lovely Professional University, India

All the suggestions made by the referee has been complied in the second version of this article.

# Peer review: Referee response for version 1

**DOI:** 10.5256/f1000research.12984.r25931

**Published:** 2017-09-19

**Authors:** Shantibhusan Senapati

**Affiliations:** 1 Tumor Microenvironment and Animal Models Lab, Institute of Life Sciences, Bhubaneswar, Odisha, India

With this review, the author provide a concise literature summary of microRNAs in the development and neoplasia of the mammary gland. For the most part the review is complete and informative. To further improve the quality, the author is advised to address these minor points:

1. Exosomes secreted by different cells of mammary gland carry microRNA. It has been demonstrated that cancer-associated fibroblasts release exosomal microRNA that induce aggressiveness in breast cancer cells (e.g. Donnarumma et al. (2017) ^1^). At the same time, the microRNA present in the exosomes secreted by the cancer cells also play a critical role in the pathogenesis of breast cancer (e.g. Melo et al. (2014) ^2^). Hence, the authors might consider to include these information.
2. The beginning sentence under “miRs in mammary gland” heading needs to be restructured. The presence of miRNA in milk and the role of miRNA in mammary development should be mentioned separately.
3. In the abstract, the sentence “Further studies in this area will help us understand ……” should be “Further studies in this area will help us to understand ……”).

I have read this submission. I believe that I have an appropriate level of expertise to confirm that it is of an acceptable scientific standard.

## References

[1] DonnarummaEFioreDNappaMRoscignoGAdamoAIaboniMRussoVAffinitoAPuotiIQuintavalleCRienzoAPiscuoglioSThomasRCondorelliG: Cancer-associated fibroblasts release exosomal microRNAs that dictate an aggressive phenotype in breast cancer. *Oncotarget*.2017;8(12) : 10.18632/oncotarget.14752 19592-19608 10.18632/oncotarget.14752 28121625PMC5386708

[2] MeloSASugimotoHO'ConnellJTKatoNVillanuevaAVidalAQiuLVitkinEPerelmanLTMeloCALucciAIvanCCalinGAKalluriR: Cancer exosomes perform cell-independent microRNA biogenesis and promote tumorigenesis. *Cancer Cell*.2014;26(5) : 10.1016/j.ccell.2014.09.005 707-21 10.1016/j.ccell.2014.09.005 25446899PMC4254633

# Response

**Published:** 2017-09-22

**Authors:** Manoj Jena

**Affiliations:** Lovely Professional University, India

To be incorporated in the heading "miRs controlling involution and breast cancer": Exosomes are cell - derived small vesicles (40 - 100 nm) containing mRNA, protein, miR etc. They are crucial mediator of inter cellular signaling during cancer development. Exosomes derived from cancer associated fibroblasts in breast cancer have 3 miRs such as miR-21, -143, and -378e which promote stemness, epithelial-mesenchymal transition, and anchorage-independent cell growth, thus accelerating oncogenic signaling in breast cancer cells (Donnarumma et al., 2017). The exosomes released from cancer cells of breast cancer patients induce the non-tumorigenic epithelial cells to form tumors with the help of dicer endonuclease (Melo et al., 2014).

The beginning sentence under “miRs in mammary gland” will be restructured. The presence of miRNA in milk and the role of miRNA in mammary development will be separated.

In the abstract, the sentence “Further studies in this area will help us understand ……” will be rectified as “Further studies in this area will help us to understand ……”.

# Peer review: Referee response for version 1

**DOI:** 10.5256/f1000research.12984.r24612

**Published:** 2017-08-07

**Authors:** Mohammed Alsaweed

**Affiliations:** 1 College of Applied Medical Sciences, Majmaah University, Al Majma'ah, Saudi Arabia

The review entitled "MicroRNAs in the development and neoplasia of the mammary gland" has briefly presented recent studies in the relevant studies in mammary gland microRNAs field. This kind of review is very good for scientists to update their knowledge without reading informative reviews. However, minor comments are below to make the review suitable for indexing:

 Introduction:

- More information is needed to understand the mechanism of microRNA roles in the mammary gland.

 

 miRs in mammary gland:

- Along with Alsaweed et al. study, Modepalli et al. (2014, BMC Genomics) has provided evidence that mammary gland is the main source of microRNAs in milk. Therefore, milk microRNAs are not considered as nutrients as stated, so better to not use "whether they are only simple nutrients".
- Examples about the milk microRNAs are important as the mammary gland is synthesized and secreted milk.

miRs controlling lactation:

- Highly expressed microRNAs in milk/mammary gland have different roles in synthesize various milk nutrient contents such as triacylglycerols and lactose, so the role of highly expressed microRNAs must be linked to related functions (Alsaweed et al 2016, PLoS ONE and IJMS).
- Li et al. (2012, BMC Genomics) has presented very important aspects of the microRNAs and lactation performance.

 

 miRs controlling involution and breast cancer:

- Zhang et al. (2013, Nature Cell Biology) has provided breast cancer inhibits progression by microRNAs expression roles, so it must be considered in this review.

I have read this submission. I believe that I have an appropriate level of expertise to confirm that it is of an acceptable scientific standard, however I have significant reservations, as outlined above.

# Response

**Published:** 2017-08-09

**Authors:** Manoj Jena

**Affiliations:** Lovely Professional University, India

**Introduction**

Mechanism of action to be incorporated: Matured miRs integrate into the RNA - induced silencing complex (RISC) and direct it to target mRNAs through partial sequence complementarity. miRs down regulate the target gene expression by translation repression (48 % through RISC complex), or mRNA degradation (29 %), or by both phenomena (23 %). (Jin and Xiao, 2015).

**miRs in mammary glands**

To be removed: The role of miRs........still disputed.

To be incorporated: miRs in milk during lactation are putative markers of activities in mammary gland and act as functional signals for proper growth and development of the young. The highly abundant miRs in milk during lactation cycle are miR-191, 184, 181, 148, 375, and miRs of let-7 family (7f, 7a and 7i) as evidenced from studies in the marsupial tammar wallaby (Modepalli et al., 2014).  

**miRs controlling lactation**

To be incorporated: Lin et al (2013) observed group of miRs (such as miR-23a, 27b, 103, and 200a) affect milk fat synthesis with synergistic action. miR profiling of lactating and nonlactating bovine mammary gland revealed miR-125b, 181a, and 199b expression level reduced in non-lactation period; whereas miR-141, 484, and 500 expression level was higher in lactation period. (Li et al., 2012). Highly expressed miRs such as let-7f-5p is found to target many genes involved in protein, carbohydrate, and triglyceride synthesis. The human milk enriched miR such as miR-22-3P regulates development and differentiation of T-lymphocytes. The miR-181a-5p and miR-182-5p have crucial role in immune cell differentiation, miR-375 is required for glucose homeostasis, and miR-148a-3p is a tumour suppressor and involved in liver development (Alsaweed et al., 2016).

**miRs controlling involution and breast cancer** 

To be incorporated: The miR 126/126* pair is observed to repress the recruitment of mesenchymal stem cells and inflammatory monocytes into the tumour stroma, thus inhibiting breast cancer metastasis (Zhang et al., 2013).
